# Digital neuropathology of neurodegenerative disorders: Foundations, research advances, and future directions

**DOI:** 10.1002/alz.70775

**Published:** 2025-11-07

**Authors:** Aaron M. Rosado, Juan C. Vizcarra, Shivam R. Rai Sharma, Cristopher Dirk Keene, Charles L. White, Ain Kim, Shelley L. Forrest, Gabor G. Kovacs, Chen‐Nee Chuah, Margaret E. Flanagan, Thomas M. Pearce, Brittany N. Dugger, David A. Gutman

**Affiliations:** ^1^ Emory University School of Medicine Department of Laboratory Medicine and Pathology Emory University Hospital Atlanta Georgia USA; ^2^ Davis Department of Computer Science University of California Davis California USA; ^3^ Department of Laboratory Medicine and Pathology University of Washington Seattle Washington USA; ^4^ Department of Pathology University of Texas Southwestern Medical Center Dallas Texas USA; ^5^ Tanz Centre for Research in Neurodegenerative Diseases, Temerty Faculty of Medicine, 1 King's College Circle University of Toronto Toronto Ontario Canada; ^6^ Davis Department of Electrical and Computer Engineering University of California Davis California USA; ^7^ Department of Pathology and Laboratory Medicine University of Texas Health Science Center San Antonio Texas USA; ^8^ Department of Pathology University of Pittsburg School of Medicine Pittsburgh Pennsylvania USA; ^9^ Davis Department of Pathology and Laboratory Medicine University of California Sacramento California USA

**Keywords:** Alzheimer disease (AD), Alzheimer's disease and related dementias (ADRD), artificial intelligence (AI), brain banking, cross‐institutional collaboration, computer vision (CV), deep learning (DL), digital pathology, medical imaging, information technology, machine learning (ML), National Alzheimer's Coordinating Center, neurodegeneration, whole slide image (WSI)

## Abstract

A neuropathology examination after death remains the gold standard for differentiating between Alzheimer disease (AD) and AD and related dementias (ADRD). Increasing interest and familiarity with digital imaging highlights recent shifts to modernize pathology workflows by leveraging technology that automates imaging and analysis. This review provides an overview of digital pathology technologies and their associated infrastructure, available open‐source and proprietary digital pathology software, relevant background on neurodegenerative histopathological features, and computational research. It further examines recent developments in digital pathology in neurodegenerative disease research with an emphasis on machine learning. We discuss evidence supporting how recently developed technologies and methodologies can enhance our understanding of histopathologic features of neurodegeneration and correlations of histopathologic features with cognitive performance and age at death. Finally, we review potential directions for neurodegenerative disease digital pathology research given trends in technological infrastructure development and other digital pathology research.

**Highlights:**

- Provides a historical summary of digital pathology with respect to neuropathology.
- Examines key digital pathology technologies.
- Explores digital pathology applications in neurodegenerative disease and their contribution to research.
- Discusses the future of digital neuropathology.

## 1 INTRODUCTION

Neuropathology is a specialized field of pathology involving the examination of nervous system tissue from either surgical biopsies or autopsies. Neuropathology is fundamental to understanding Alzheimer disease (AD) and AD and related dementias (ADRD) and is considered the diagnostic gold standard. The logistics of sharing valuable tissue specimens acquired at autopsy can make external research access difficult. With the advent of slide scanning technology, digitization of histologic sections provides more efficient means of distributing these rich data sources and enables sophisticated quantitative analytical approaches. Digitization alone, however, does not immediately provide a development pathway for robust, quantifiable, reproducible, and generalizable brain tissue analysis pipelines. Many individual steps exist between initial tissue collection, processing, sectioning, and staining prior to digitizing each sample. Large and complex microscopic imaging data also require highly specialized training and significant person‐hours for neuropathological sample analysis, which can hinder research progress. Despite these challenges, improvements in digital pathology instrumentation and software tools are rapidly progressing, offering neurodegenerative disease researchers powerful tools to leverage in their investigations.

This manuscript reviews key digital pathology technologies and their associated research advancements in neuropathology. It also highlights challenges at the intersection of digital pathology and neurodegeneration research with respect to motivating collaborative efforts between computational and neurodegenerative disease researchers. Multidisciplinary collaborations utilizing digital pathology images in neurodegenerative disease research have great potential to enhance our understanding of neurodegenerative disease pathogenesis and clinical presentation.

## 2 NEUROPATHOLOGY FOUNDATIONS IN DIGITAL PATHOLOGY

### 2.1 Brief history of origins of digital pathology in neuropathology

**FIGURE 1:**
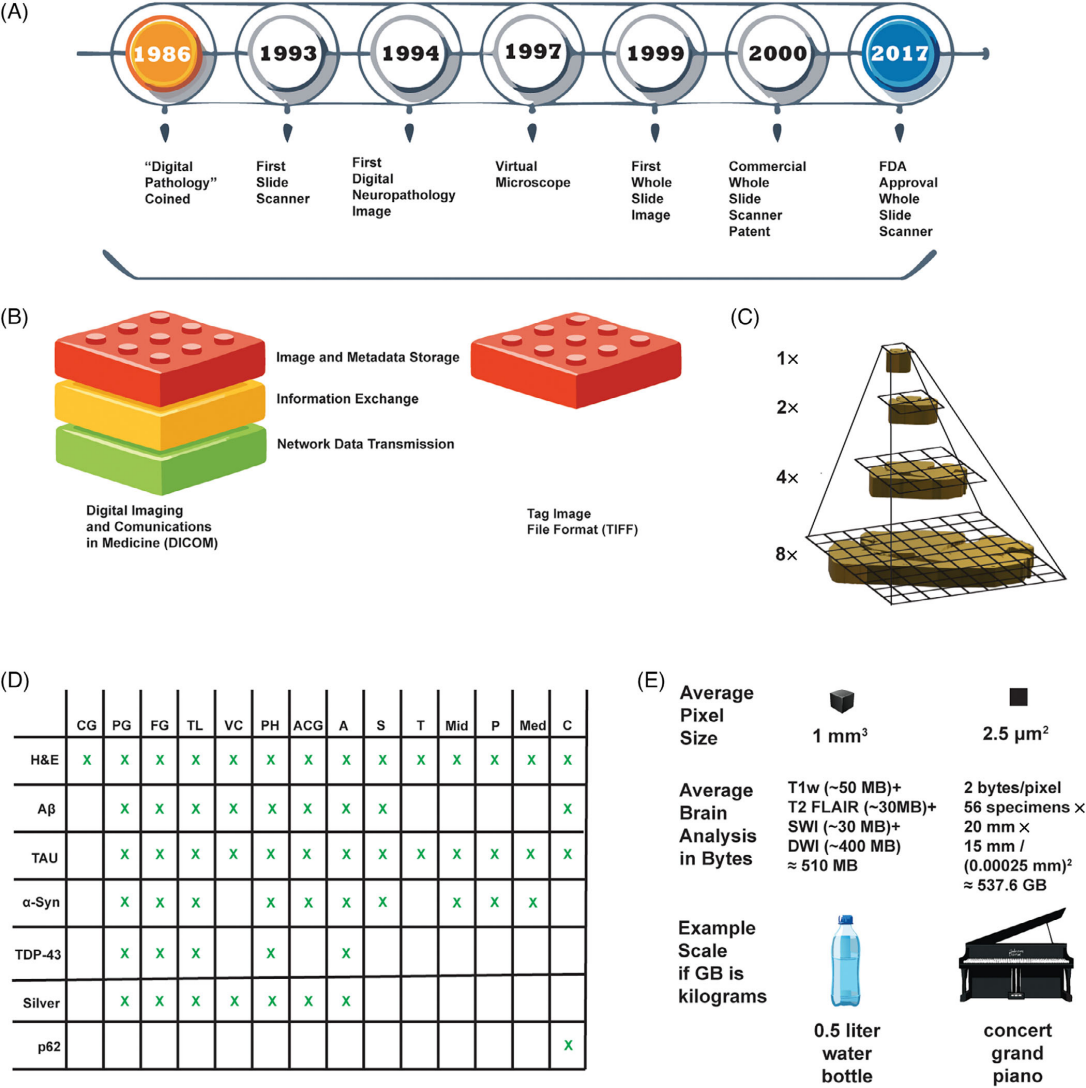
Background in digital pathology for understanding key features of related data. (A) Timeline of key events in digital pathology from coinage of original term *digital pathology* to first Food and Drug Administration (FDA) approval of a digital pathology scanner (Aperio Technologies). (B) Comparison of digital imaging and communications in medicine (DICOM) and tag image file format (TIFF) highlighting how DICOM is an open technological standard describing the storage, exchange (transfer between devices), and transmission (over a network using communication protocols) of digital medical imaging data and the TIFF format focuses on metadata and image data storage. (C) Example of a pyramidal image showing data information created from multiple magnification scales (can be physical or virtual) showing same sample increases in number of representative tiles as resolution increases. An outline of a neuropathological sample was created from the same neuropathology image featured in Figure 3 and features a hypothetical square grid created for the sample at hypothetical resolutions (1×, 2×, 4×, and 8×). (D) A table documenting typical brain anatomic regions (column headings) and stains (row headings) used by Emory University at autopsy for neurodegeneration diagnoses. Non‐immunologic stains include hematoxylin and eosin (H&E) and silver. IHC stains include amyloid beta (Aβ), tubulin‐associated unit (tau), alpha‐synuclein (α‐syn), transactive response binding protein 43 kilodaltons (TDP‐43), and sequestosome‐1 (p62). Anatomical regions: central gyri (CG), parietal gyri (PG), frontal gyri (FG), temporal lobe (TL), visual cortex (VC), posterior hippocampus (PH), anterior cingulate gyrus (ACG), amygdala (A), striatum (S), thalamus (T), midbrain (Mid), pons (P), medulla oblongata (Med), and cerebellum (C). (E) Comparison of differences in image information generation in magnetic resonance image (MRI) generated from a living person and whole slide image (WSI) performed at autopsy. We assume a typical brain MRI is performed at a resolution of 1 mm^3^ of the average volume of an adult brain (93 mm height, 167 mm length, 140 mm width) with an uncompressed image with 4 bytes per voxel (three‐dimensional pixel). For comparable WSI information, we assume a typical Emory neurodegenerative autopsy tissue processing of a single brain hemisphere with average specimen area (20 × 15 mm)^18^ and 2 bytes of data per two‐dimensional pixel create an estimated 537.6 gigabyte (GB) uncompressed image data. For a typical MRI scan we assume an analysis involving T1‐weighted (T1w), T2 fluid‐attenuated inversion recovery (T2 FLAIR), susceptibility weighted image (SWI), and diffusion‐weighted MRI (DWI) sequences total approximately 510 megabytes (MB).^19^ If gigabytes were equivalent to kilograms, an example comparison in data quantity would be a 0.5‐L bottle of water (0.5 kg) with respect to a concert grand piano (408–544 kg) for an MRI and WSI, respectively.^20^

Analogously to the migration from plain (analog) films to digital image files in radiology, emerging scanning technologies allow histologic samples to be digitized and stored electronically. Pathologist Ronald Weinstein inspired the term “digital pathology” (coined in 1999)^1^ ^,^ ^2^ in 1986 as the idea of telepathology emerged, involving the utilization of digital images for remote pathologic diagnosis (Figure 1A).^3^ In the early 1990s, neuropathology image digitization enabled digital image use in research and teaching, with early examples including computer‐assisted morphometric analysis of neuropil threads in AD.^4^ In 1994, James Bacus built the first commercial slide scanner, a digital microscopy system that scanned a single standard microscope slide in 24 hours.^5^ The applied principles behind slide scanning created the first virtual microscope by initially capturing a large specimen image using robotic instrumentation.^6^ The first whole slide image (WSI) was created using an automated high‐speed system in 1999,^7^ leading to the first commercial whole slide scanning instrumentation in the early 2000s.^8^, ^9^, ^10^ Digital pathology adoption increased markedly in the 2010s in efforts to optimize diagnostic processes.^11^ Some academic institutions with digital pathology capabilities elected to create WSI repositories for education and research. Academic interest in digital pathology motivated open‐source software development for reading and writing WSIs.^12^, ^13^, ^14^ In 2017, U.S. Food and Drug Administration (FDA) approval for WSI technology enabled clinical adoption for primary pathology diagnosis in a surgical pathology context.^15^ ^,^ ^16^ Concurrently, adoption of machine learning (ML) and artificial intelligence (AI) technologies provided new and powerful image analysis capabilities for pathologic specimens, including neuropathologic samples.^17^

### 2.2 Key digital pathology technologies

Since the creation of the first commercial slide scanner in 1994, several instrument companies have developed their own slide scanners with features motivated by clinical, educational, and research needs. High‐throughput scanners utilize slide handling machinery, mechanical microscope stage manipulation, objective handling, as well as high‐quality optics and sensors to efficiently and rapidly acquire digital images for in silico combination into larger images by integrating lower‐ and higher‐magnification fields (typically at 20× or 40× magnification). Modern scanners can scan an entire 25 × 75 mm (1 × 3 inch) glass slide in 1 to 2 min.^21^, ^22^ Many slide scanner manufacturers offer high‐quality image generation from slide batches at speeds suitable for large‐scale clinical and research applications, with some manufacturers achieving FDA approval for use in clinical diagnosis. Likewise, various commercial products claim suitability for research and clinical applications including but not limited to Leica Biosystems Aperio series, Philips Digital Pathology Solutions IntelliSite series, Hamamatsu Photonics Nanozoomer series, Nikon BioPipeline Slide, 3DHISTECH PANNORAMIC series, Roche Ventana series, Zeiss Axioscan, Olympus SLIDEVIEW, Motic Digital Pathology MoticEasyScan series, Huron Digital Pathology TissueScope series, and Grundium Ocus series.^8^ WSI scanners support rapid brightfield and, less commonly, fluorescent image acquisition, depending on the specific make and model.^23^ Notably, neurodegeneration and magnetic resonance image (MRI) correlation studies benefit from imaging larger tissue specimens such as whole cerebral hemispheric sections and building an image z‐stack for three‐dimensional histologic image creation,^24^ a feature found on certain scanners such as Huron Digital Pathology TissueScope LE.^25^ In addition to hardware differences, manufacturers also vary image information and associated metadata representations in WSIs. Associated metadata can refer to information about the slide scanner used to acquire the image, properties describing digital and physical image properties, and patient‐related information.^26^ Proprietary software refers to software in which an individual or commercial entity owns exclusive rights that restrict its use, source code inspection, source code modification, and redistribution.^27^ Unlike radiology's digital imaging and communication (DICOM) technical standard for digital storage and transmission, most WSI manufacturers use proprietary image file formats that can hinder data sharing and analysis across scanners/image types.^28^ WSI image files are large, and files created from glass slides for a single patient/individual create tremendous amounts of imaging data. Before compression, digitized histologic slides of a case using a standard neurodegenerative workup such as that used by Emory University (Figure 1D) can generate upwards of 500 gigabytes (GB) of data, which is orders of magnitude more than the 500+ megabytes (MB) for a typical MRI brain study (Figure 1E).^29^ ^,^ ^30^ Despite proprietary manufacturer file formats, WSI image files are built upon common digital image technologies such as joint photographic experts group (JPEG) and tag image file format (TIFF), with the most widely adapted technology being TIFF (Figure 1B). In designing their respective file formats, manufacturers vary image compression, image data organization, and associated metadata.^12^, ^21^, ^31^ WSIs often utilize a pyramidal information structure to encode digital image pieces commonly referred to as “tiles” that allow performant software (Figure 1C).^32^ Most U.S.‐based brain banks, including those within AD research centers, reported in a 2019 survey that Aperio (.svs) was the most common file format used.^33^ Other notable file formats used include Hamamatsu (.ndpi), Philips (.tiff), and Zeiss (.czi), with Aperio and Philips both being TIFF variants.^28^

Open source refers to software where the instructions, referred to as source, defining the software's functionality are openly available.^27^ Open‐source efforts also exist for creating a standardized WSI format such as Open Microscopy Environment (OME)‐TIFF, whose developers also provide a complete reading/writing library with tools for converting proprietary formats.^28^ Open‐source software for reading proprietary WSIs include openslide, bio‐formats, and large image.^12^ ^,^ ^14^ ^,^ ^31^, ^34^ While a WSI‐DICOM format has been developed its adoption by vendors is not yet universal perhaps because the format needs to be natively created by WSI slide scanners, demonstrate acceptable performance in practice that keeps up with demanding slide scanning rates, and be supported by all downstream image management and third‐party solutions used.^35^, ^36^, ^37^

Many vendors have developed their own commercial image platforms to store, manage, and retrieve WSI clinical data. Examples of slide scanner manufacturer commercial image platforms include Phillips IntelliSite Pathology Solution, Leica Biosystems Aperio eSlideManager, Hamamatsu NDP.serve3, and Roche Ventana Virtuoso. Several companies also established vendor‐agnostic platforms such as Proscia (Concentriq), Indica Labs (HALO), and Sectra's Digital Pathology Solution. Slide scanner manufacturer commercial image platforms address challenges in networking, security, Health Insurance Portability and Accountability Act (HIPAA) auditing, and archiving capabilities essential for pathology diagnosis. Many commercial platforms also offer integrations with AI‐based analysis and assistant tools into pathology workflows.

### 2.3 Adoption challenges

Although interest in digital pathology is increasing in neuropathology, key barriers remain that impede its widespread adoption. Many brain biorepositories have been collecting and processing tissue years or even decades prior to the advent of digital scanners; the first brain collections for studying neurologic and psychiatric diseases were initiated by neuropathologist John Arthur Nicholas Corsellis in 1951 and Wallace Tourtellotte's National Neurological Research Bank in 1961.^38^ ^,^ ^39^ Since then, institutions have adopted various staining and blocking protocols based on institutional needs and research foci.^29^, ^33^

Within the United States, neurodegenerative disease digital pathology data have primarily come from AD Research Centers (ADRCs), where samples are processed and stored with associated metadata based on uniform data collection guidelines set forth by the National Alzheimer's Coordinator Center (NACC).^40^, ^41^ Currently, not all ADRCs have access to slide scanners.^33^ FDA approval likely shapes access to slide scanning hardware and digital pathology image management systems because FDA validation ensures potential use beyond research applications.^42^ Access to clinical‐grade slide scanners and digital pathology image management systems also remains limited given the substantial cost, necessary additional information technology infrastructure and regulatory oversight, and need to support dedicated personnel to transition and support the platform.^43^ For those ADRCs that digitize slides, it is optimal for WSIs to be linked to metadata from the brain bank itself as well as relevant clinical data to be maximally useful. Many brain biorepositories store vital metadata using spreadsheet software; enhanced data management systems are needed that bridge spreadsheets with digital pathology image management systems and other multimodal databases such as, but not limited to, demographics, family history, medications, disease‐specific treatments, health history, neurological examination, clinical symptom judgement, neuropsychological battery scores, and related clinical assessment tools (Unified Parkinson's Disease Rating Scale, Clinical Dementia Rating [CDR] Dementia Staging Instrument Plus NACC Frontal Temporal Dementia Behavior and Language Domains, Neuropsychiatric Inventory Scale, Geriatric Depression Scale, and NACC Functional Assessment Scale).^44^ Many of these data are captured within the NACC database and associated ADRCs.^40^ ^,^ ^45^, ^46^, ^47^, ^48^, ^49^

The resources needed for such an endeavor are substantial, particularly for clinical use. As an example, estimated infrastructure costs for a large institution such as Memorial Sloan Kettering (MSK) between 2006 and 2021 include $370,000 on infrastructure planning staff, $7.5 million on slide scanners, $1.6 million in maintenance costs, $1.6 million in onsite data storage, and $630,000/year in software‐related costs for internal tool development and maintenance.^50^ Despite these costs, MSK found a significant amount of cost savings by reducing physical microscope slide handling and estimated their cost per slide between $0.55 and $19.53 depending on throughput utilization. Notably, MSK's analysis publication highlights intangible financial benefits in attracting key faculty and staff in supporting institutional research and development. Key elements from MSK's analysis include their use of internal information technology (IT) resources, which are likely an important source of cost savings given cloud‐based platforms are commonly designed for users with lower throughputs in data transfer and storage unlike WSI infrastructure needs. Additional research into costs and savings in neuropathology for neurodegenerative diseases could aid in efficient integration into existing institutional WSI resources and developing independent infrastructure provided a converting institution already has infrastructure in place. For example, institutions supporting digital pathology‐based cancer diagnosis create specimens from various biopsies involving blood or tissue samples collected from living humans that necessitate certain standards and quality control measures based on clinical diagnosis needs^51^ that diverge from ADRC brain specimen banking. A 2023 survey reported ADRCs collected brain samples from either one or both brain hemispheres in 19 anatomic regions with variable staining protocols, demonstrating digital pathology research would generate a large collection of WSIs for histopathological characterization.^29^ Given this potentially large WSI collection, reduced sample handling and increased productivity could also benefit neurodegeneration research.

## 3 DIGITAL PATHOLOGY TOOLS

### 3.1 Visualization and annotation tools

WSI data visualization and annotation require software capable of interpreting image and metadata information stored in files that are often proprietary. As previously stated, manufacturers sell and/or make freely available commercial versions of their software to facilitate working with WSIs generated by their slide scanners. Despite their availability, limitations in the functionality of such software for common research use cases motivated the development of several commercial and open‐source products designed to access and visualize WSI data, assist analysis, and integrate computational analysis tools.^52^ Software written for visualization and annotation should allow for simple access to WSI metadata, facilitate visualization of various regions within a file at different magnifications, enable drawing representative shapes that can be mapped to specific places within an image, and ideally export such information in a common format for use in other software contexts. Such information could be easily saved and associated with the original WSI in such a way as to simplify data generation and provide a basis for re‐analysis or additional analysis. Visualization and annotation features allow for aggregating relevant qualitative and quantitative data from multiple images depending on investigator hypothesis. Collecting relevant information for pathological diagnosis and research from WSIs serve as a foundation for downstream applications in ML. Table 1 features a non‐exhaustive list of open‐source and proprietary tools highlighting support for common WSI file formats, native and/or third‐party analysis support, and associated FDA approval aggregated from an internet search. Although not required for neurodegeneration or other digital pathology research, FDA oversight is mentioned given its influence on institutional availability and adoption.

**TABLE 1:** Digital pathology visualization and analysis tools.

Name	Analysis tools	Supported formats	Open source	Platform	Clinical workflow support	FDA approval status as of April 2025
HALO^53^	AI‐based image analysis, automated multiplex IHC, spatial biology, tissue classification, object detection, quantitative scoring	SVS, TIFF, NDPI, VSI, DCM (DICOM), MRXS, ND2, VSI, SCN, LIF, BIF	No	Windows, macOS	Yes	Partial^54^
Visiopharm^55^	Advanced AI model builder, phenotyping, tissue segmentation, stereology, biomarker quantification	SVS, TIFF, NDPI, DICOM	No	Not listed	Yes	Partial
PathAI AISight^56^	Proprietary deep learning models for diagnostic support, cancer grading, inflammation detection	TIF, MRXS, DICOM	No	Not listed	Yes	Partial
PathPresenter^57^	Interactive pathology image viewer, telepathology, educational overlays	Not listed	No	Web‐based	Yes	Partial
Zegami^58^	Visual data exploration, clustering, annotation, image‐based AI training	Not listed	No	Web‐based	Not available	Research only
Corista DP3^59^	Workflow automation, AI plugin integration, diagnostic reporting, image routing	DICOM	No	Web‐based	Yes	Partial (pending)
Roche navify^60^, ^61^	Cloud‐based digital pathology with AI‐enabled decision support and workflow integration	TIF, DICOM	No	Web‐based	Yes	Partial
Aiforia^62^	Deep learning model builder, cloud deployment, multiplex analysis, image classification	Not listed	No	Web‐based	Yes	Partial
Philips IntelliSite pathology solution^63^	FDA‐cleared WSI viewer with iSyntax format; integrates with laboratory information system; AI support modules	iSyntax, SVS, MRXS, NDPI, TIFF^63^	No	Web‐based, Windows	No	FDA‐cleared for primary diagnosis
Leica Aperio eSlide Manager & ImageScope^64^	High‐throughput slide manager with AI integration from Paige for prostate/breast	SVS, QPTIFF, NDPIS, MRXS	No	Web‐based, windows	No	FDA‐cleared for primary diagnosis
Paige Platform (Full Focus)^65^	AI‐assisted diagnosis for cancer; includes Paige prostate suite	SVS, NDPI, DICOM	No	Windows, macOS	No	FDA‐cleared for AI‐assisted WSI diagnosis in prostate cancer
Ibex Galen platform^66^, ^67^	AI for cancer detection and grading; real‐time alerts; Conformité Européenne in vitro diagnostic compliant	iSyntax	No	Windows, MacOS	No	FDA‐cleared for AI‐assisted WSI diagnosis in prostate and breast cancer
QuPath^68^, ^69^	Open‐source bioimage analysis, batch scripting, cell segmentation, ML classifiers	TIFF, SVS, NDPI, MRXS	Yes	Windows, macOS, Linux	No	Research only
Orbit image analysis^70^	ML platform for histology pattern recognition; custom classifier training	TIFF, OME‐TIFF, SVS, NDPI, CZI, DICOM (via bio‐formats)^71^	Yes	Windows, macOS, Linux	No	Research only
SlideRunner^72^	Manual annotation tool for ML training; supports crowd‐sourced labeling	TIFF, DICOM (via openslide)	Yes	Windows, macOS	No	Research only
Automated slide analysis platform^73^	OpenSlide‐based viewer with ML integration, tiling, batch processing	TIFF, SVS, NDPI, DCM (DICOM) (via openslide)	Yes	Windows, Linux	No	Research only
Digital Slide Archive^74^	Federated analysis, plugin‐ready AI models, pathology image archive, visualization	SVS, NDPI, TIFF, MRXS, CZI, DCM, ND2, OME‐Zarr, OME‐NGFF, OME‐TIFF (via large image^14^)	Yes	Web‐based	Yes	Partial
Open Microscopy Environment OMERO^75^	Central image repository, metadata linking, multi‐user access, integration with tools	TIFF, OME‐TIFF, SVS, NDPI, CZI, DICOM (via bio‐formats)	Yes	Windows, macOS, Linux	No	Research only
Grand challenge^76^	Benchmarking platform for AI algorithm validation and comparison	SVS, NDPI, TIFF, MRX, DCM (DICOM)	Yes	Web‐based	No	Research only

*Note*: Google and OpenAI GPT‐4o were used to generate a list of visualization and analysis tools (last updated on May 31, 2025) available for digital pathology highlighting the availability of AI‐based analysis, supported file formats, supported platforms (operating system versus web‐browser), support for clinical workflows, and FDA approval. FDA approval is highlighted given its influence on institutional availability, adoption, and availability of relevant information documenting operating system and file format support. If data regarding supported formats could not be obtained from manufacturers’ public websites, the information was obtained from FDA 510k submissions. The term AI refers to the broader computer vision definition and does not imply statistical or subsymbolic learning. AI analysis refers to tools built into the visualization/analysis tools, while AI integration refers to AI capabilities supported by first‐ and/or third‐party externally run software. AI model training implies the use of data and a systematic process that enable a computer to estimate desired outputs for a specific task from input data most commonly by optimization algorithms such as stochastic gradient descent. These software products might have changes in platform support and functionality as many are still in active software development.

Abbreviations: AI, Artificial Intelligence; BIF, BioImagene Image File; CZI, Carl Zeiss Image; DCM, DICOM file; DICOM, Digital Imaging and Communications in Medicine; DP3, Digital Pathology Processing Platform, FDA, Federal Drug Administration; GPT, Generative Pre‐trained Transformer; IHC, Immunohistochemistry; LIF, Leica Image Format; ML, Machine Learning; MRXS, MIRAX Virtual Slide Format; ND2, N‐Dimensional Image; NDPI, NanoZoomer Digital Pathology Image; NDPIS, NanoZoomer Digital Pathology Image Stained; NGFF, Next Generation File Format; OME, Open Microscopy Environment; OS, Operating System; QPTIFF, Quantitative Pathology Tagged Image File Format; SCN, Scene file; SVS, ScanScope Virtual Slide; TIFF, Tagged Image File Format; VSI, Virtual Slide Image.

Additional proprietary software visualization and annotation tools have emerged seeking to fill gaps in manufacturer image management systems aiming to provide enhanced analysis functionality. As listed in Table 1, key features for visualization and annotation platforms include ML analysis tool integration and model training capabilities aimed at workflow improvement. Most proprietary visualization and annotation tools support Windows (based on National Cancer Institute or FDA requirements) or web browsers based on information acquired from publicly available software, product pages, and public FDA data (Table 1). Only proprietary platforms are currently FDA cleared either partially or for primary diagnosis given the significant overhead and costs required for clinical validation and Health Insurance Portability and Accountability Act compliance (Table 1). As of May 2025, only the Leica Aperio eSlide Manager with Image Scope viewer and Phillips IntelliSite Pathology Solution are fully FDA compliant for primary diagnosis (Table 1); the Paige Platform and Ibex Galen Platform are the only FDA‐cleared AI‐assisted WSI diagnosis tools.^66^, ^77^ HALO, Visiopharm, PathPresenter, and Aiforia are visualization and annotation tools featuring integrated ML tools with partial FDA clearance primarily used in clinical research, though these are expanding into the clinical space as well (Table 1).

Open‐source alternatives to proprietary visualization and annotation tools exist. Open‐source software features many different licenses governing its legal use allowing, limiting, and/or prohibiting its use in commercial products in ways that shape their profitability. Open‐source software projects generally seek community input on functionality and problems and encourage developers to contribute to source code development, although often open‐source projects can be supported primarily by commercial investments.^78^ Notable open‐source visualization platforms for digital pathology include QuPath and the Digital Slide Archive (DSA) (NIH U24‐CA194362‐01),^69^, ^74^ which support many WSI file formats with the DSA enabling clinical workflows. Most open‐source tools focus on supporting ML research offering tools to integrate existing ML tools/workflows, inspect their output, and utilize data in training novel ML models. Open‐source software projects typically support more operating system (OS) platforms, including MacOS and Linux, with the DSA notably being a web‐based platform supporting a large file format collection (Table 1). The DSA is an open‐source product maintained by the technology company Kitware, Inc. utilizing its large‐image and girder products to provide a web interface that integrates multiple WSI viewers including OpenSeadragon, a web‐based viewer for high‐resolution zoomable images built in pure JavaScript.^79^

### 3.2 Deidentification tools

Removing protected health information (PHI) from WSIs generated for diagnoses and/or research purposes is often a critical step before the data can be made more openly available to trainees and researchers. Deidentification tools must understand typical filenames, metadata, associated images, and annotations of or included in common clinical WSI file formats, allow for the removal of PHI stored within all fields, and maintain useful research metadata.^80^ ^,^ ^81^ Open‐source deidentification tools include SVS‐deidentifier, Medical Image De‐identification tool, ImageDePHI, DSA WSI DeID, anonymize‐slide, and DICOM Standardization tools. SVS‐deidentifier focuses on the Aperio SVS file format supporting PHI removal through a Python‐based application with a browser‐based front end.^82^ ImageDePHI is an open‐source command line‐based tool primarily supporting PHI redaction from Aperio SVS and pure tiff files.^83^ Kitware's DSA WSI DeID enables anonymization using a DSA‐based workflow.^84^ Anonymize‐slide was designed to removal labels from Aperio SVS, Hamamatsu NDPI, and 3DHISTECH MRXS.^85^ Holub et al. 2023 developed a privacy risk analysis model for identity disclosure from histopathologic images seeking to guide researchers in understanding the risks associated with using real‐world imaging data and provide guidelines for minimizing risks associated with sharing WSI data. Ideally, a user‐friendly open‐source deidentification tool will emerge supporting all clinical WSI file formats. Currently, a team, led by Dr. Gutman and Dr. Pearce at Emory University and the University of Pittsburgh, respectively, is developing a desktop‐based deidentification tool using the Electron application framework that will combine a simple user interface for deidentifying large WSI collections and streamlining deidentified data sharing through a DSA‐based platform, the Brain Digital Slide Archive (BDSA).^86^

### 3.3 Data aggregation and sharing

**FIGURE 2:**
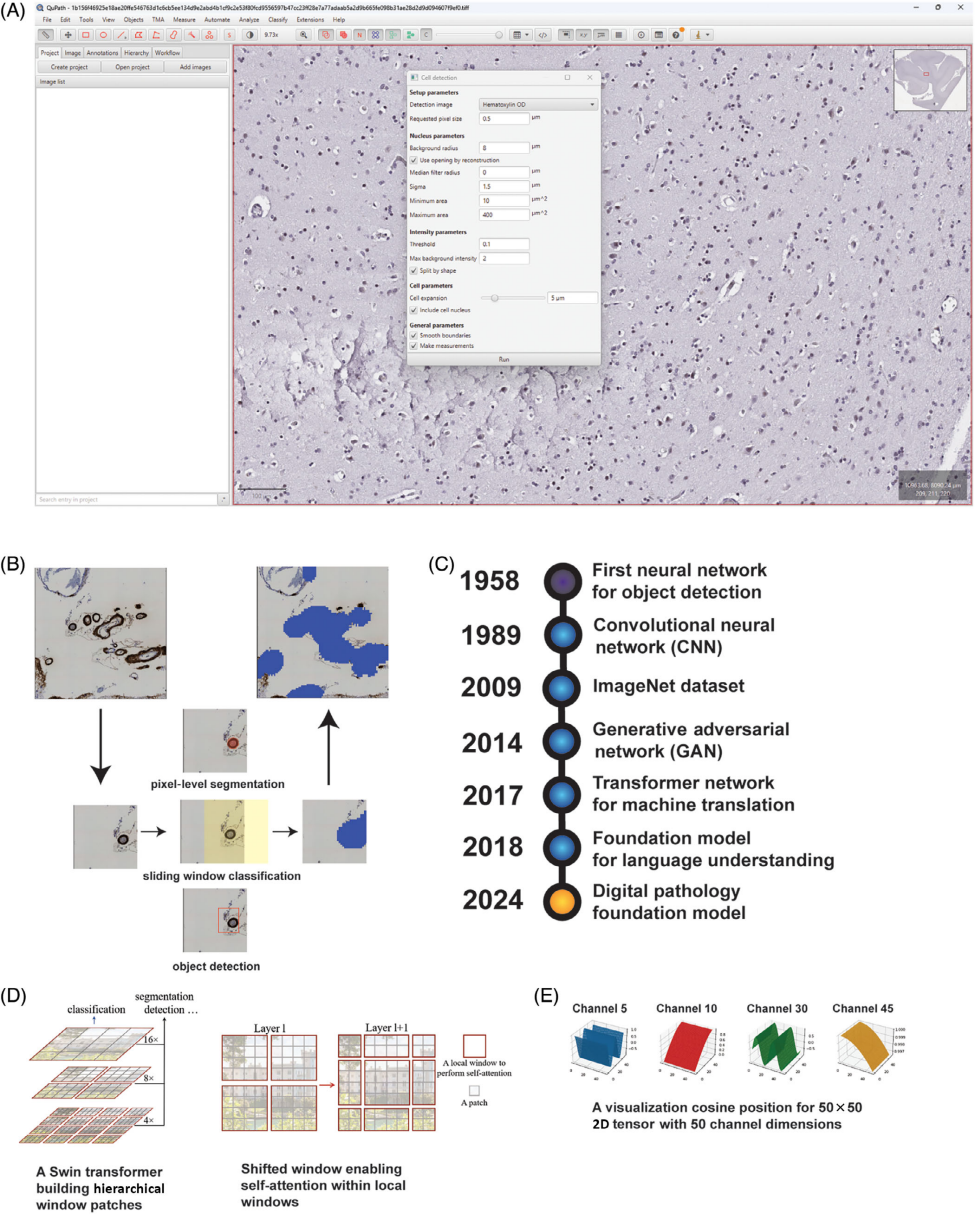
Relevant technologies in study of neurodegenerative disease in neuropathology. (A) Screenshot of QuPath^69^ for digital pathology analysis featuring a deidentified immunohistochemistry (IHC) image in the tagged image file format (TIFF) showing an interface for cell detection enabling size‐based thresholds for relevant areas, intensity, and exclusion/inclusion of the cell's nucleus. (B) Visualization of automated pathology quantification in IHC‐stained WSI tile containing multiple cerebral amyloid angiopathy (CAA) deposits using a segmentation model to predict pathology boundaries, object detection to predict pathology enclosing bounding box coordinate, and sliding window classification to classify overlapping regions that delineate pathology boundaries.^88^, ^89^, ^90^, ^91^ Sliding window‐based processing gets pixel‐level CAA boundaries (brown staining in IHC image) and then constructs an annotation mask (blue pixels). (C) Timeline of instrumental events in development of neural networks leading to most recent development of digital pathology foundation model.^92^, ^93^, ^94^, ^95^, ^96^, ^97^ (D) Swin transformer utilizing transformer‐based attention to interpret image patches and subsequent patch groupings generated from shifted windows highlighting how attention‐based mechanisms can influence transformer performance in relevant image tasks (classification, segmentation, detection). Figure image was directly copied from Swin transformer repository with minimal modifications for improving text visualization with permission for reuse obtained from Baining Guo.^98^ (E) Visual representation of cosine‐positional embedding for a two‐dimensional 50 × 50 tensor with 50 channels. Image copied directly from Peter Tatkowski's GitHub repository with minimal modifications to improve text visualization^99^ after obtaining his permission over GitHub.

Data aggregation and sharing platforms allow easy access to WSI data, metadata, and various analysis outputs. The previously mentioned commercial platforms provide many of the core functionalities needed for research and development, but often their proprietary nature hinders research and application development. Proprietary platforms often limit data access and analysis accessibility to protect proprietary intellectual property, that is, the black box of how the data were accessed and the exact parameters of the analysis done. Applications typically use an Application Programming Interface (API) that enables querying specific data elements to simplify building a complex application. Given the speed and quality of progress made by computational researchers who build openly available frameworks and tools, slide scanner manufacturer commercial platforms remain limited in their ability to keep pace with research labs and software companies who utilize and develop open‐source tools.^52^ To address the digital pathology research community needs, manufacturers like Phillips began focusing on developing open platforms to accelerate research and development.^87^ Open platforms typically focus on accessible Representational State Transfer (REST) APIs, a common tool used by software developers to integrate data and functionality from useful platforms into their software. For example, REST APIs simplify common data operations such as accessing, updating, and deleting WSI‐related data. Openly accessible platforms will likely become more common among manufacturers as they attempt to leverage open‐source research projects and models to increase useful analysis tool accessibility for their users. Examples of open‐source image platforms include the DSA and Open Microscopy Environment OMERO (Figure 2B). Notably, the DSA's open‐source code base allows for development flexibility by allowing researchers to extend core REST API functionality for their specific use cases. The DSA's REST API simplicity as well as accessible Python and JavaScript API clients provide the DSA with important software tools that empower researchers. The DSA project supports data sharing for collaborative research initiatives in digital pathology research like The Cancer Genome Atlas (TCGA) through the Cancer Digital Slide Archive (CDSA) research demonstrating the platform's usefulness in research initiatives as well as connecting to other data sources such as those within NACC.^40^ ^,^ ^74^

In addition to open platforms, open data repositories such as those from NACC and the Alzheimer's Disease Neuroimaging Initiative (ADNI) aid research in specific problem domains or make research paper data public to help research community members validate findings or utilize data to answer broader research questions.^100^ ^,^ ^101^ Notably, sharing research data from papers in addition to advancing science may increase citation rate,^102^ providing additional incentives for open data sharing. Other open data repositories include Zenodo,^103^ Dryad,^104^ and Globus.^105^ Zenodo is a European Organization for Nuclear Research (CERN) open data repository built for research that maps data with Digital Object Identifiers (DOIs) to simplify citations. Zenodo limits users per research record to a total of 50 GB (as of August 27, 2025), making it suitable for around 12 large WSI files (assuming 4 GB per file).^103^ The non‐profit Dryad offers a similar solution for sharing research data associated with publications through a multi‐institutional collaboration formed between many academic and research institutions. Dryad currently supports single files that should not exceed 10 GB and limits data per publication to 2 terabytes (TB) (as of August 27, 2025), making it suitable for sharing a medium‐sized WSI collection (∼ 512 * 4‐GB WSI files).^104^ Given the large size of many research datasets, emerging non‐profit platforms like Globus focus on simplifying data and computational resource sharing among research collaborators. Globus requires users to use internal or external data storage and transfer platforms, limiting its usefulness beyond simplifying and controlling data access.^105^ Data sharing can require verifying PHI removal to share valuable research data and may necessitate significant investments to navigate legal and technical challenges. The BDSA project is currently navigating the legal and technical challenges associated with sharing large WSI collections among participating research institutions to create a large neurodegeneration WSI collection.

### 3.4 Computer vision use in neuropathology research of neurodegenerative disease

Computer vision (CV) involves using computational methods to extract useful information from digital images. CV can also refer to a field within ML utilizing statistical learning or deep learning (DL) models to interpret digital images. In the context of this review article, we refer to CV as the use of computational methods to acquire, process, analyze, and understand digital images and extract high‐dimensional data from them. Many research fields employ CV to retrieve relevant information from digital images and utilize such information to develop qualitative and quantitative data to answer research questions. For example, image intensity within a defined area (i.e., region of interest [ROI]) serves as a common quantitative measure in sample comparisons that can be programmatically obtained using CV techniques.

WSI visualization and analysis tools often make CV techniques easily accessible with well‐designed user interfaces for controlling various parameters and utilizing computational output. The most common usage typically involves computational operations that focus on classifying and subsequently quantifying pixels, the smallest image information unit contained within a digital image. For example, algorithms such as positive pixel count (PPC) may algorithmically change color representation to select positively stained pixels and quantify stained pixels within a defined image area,^106^ ^,^ ^107^ a technique that enabled quantification of amyloid beta (Aβ) plaques correlating well with AD‐related scoring schemas^108^ and demonstrated robust and reproducible measurements across different magnifications and repeated scans.^109^

QuPath offers many CV tools (Figure 2A) allowing researchers to detect tissue through thresholding techniques, defining ROI for measurements, cell detection, and classification, as well as utilizing pixel classifiers. QuPath additionally can perform more complex analysis through a scripting interface, a tool that consumes user‐defined text defining a sequence of operations to accomplish a desired task. QuPath also works with other common research CV platforms such as ImageJ and OpenCV, a commonly used digital image analysis tool in many research domains and an open‐source CV platform commonly used by software engineers for instrument and robotic software, respectively.^52^ ^,^ ^69^ ^,^ ^110^ Since 2017, there have been several examples of QuPath use in neuropathology research: automating immunohistochemistry (IHC) stain quantification in animal models of closed head injury,^111^ quantifying astrocyte responses by glial fibrillary acidic protein (GFAP) and 3‐3′‐diaminobenzidine (DAB) IHC staining in an animal model of mechanical brain injury,^112^ quantifying pericytes and microglia in a transgenic mouse model expressing fluorescent neuron‐glial antigen 2 (NG2),^113^ quantifying 3R/4R tubulin‐associated unit (tau) pathological inclusions in *post mortem* human brain tissues,^114^ quantifying tau and Aβ by IHC staining in *post mortem* human brain tissue samples acquired from AD or control subjects,^115^ quantifying p53‐binding protein 1 (53BP1) in *post mortem* human left frontal cortexes,^116^ and quantifying transactive response (TAR) DNA binding protein‐43 (TDP‐43) in *post mortem* human brains from individuals with frontotemporal dementia (FTD) with or without movement disorders.^117^

Commercial tools like Aperio ImageScope and HALO can also facilitate CV analysis tool usage for WSIs. Examples of Aperio ImageScope usage include quantifying GFAP and ionized calcium binding adaptor protein 1 (Iba‐1) burden in Aβ precursor protein (APP) transgenic mice,^118^ examining select brain stem nuclei in subjects with or without rapid eye movement sleep behavior disorder,^119^ determining phosphorylated poly‐Ub positive cell count, Lewy body dementia, and neurofibrillary tangle (NFT) count in *post mortem* human brains,^120^ quantifying AD progression by neuronal and microglial markers in a *post mortem* human brain with idiopathic normal pressure hydrocephalus,^121^ and determining NFT burden in *post mortem* human brains for downstream neural model training.^122^

Similarly, examples of HALO use include demonstrating generalizability by validating findings with QuPath^123^ and studying interactions between integral membrane protein 2B (ITM2B) and intracellular tau pathology in *post mortem* human samples with AD neuropathologic changes and limbic‐predominant age‐related TDP‐43 encephalopathy changes,^124^ determining Aβ, phosphorylated tau, Iba‐1, and GFAP burden in *post mortem* human brains acquired from presenilin 2 (PSEN2) N1411 variant and unrelated donors with Down syndrome,^125^ and conducting quantitative neuropathologic analysis of superior and middle temporal gyrus in *post mortem* human brains that underwent the rapid procedure (optimized tissue collection, slicing, and freezing) chosen to represent the full spectrum of AD neuropathology with or without comorbid age‐related pathologies.^126^

### 3.5 ML: history and use in medical imaging

AI is a field of research focused on developing computer systems capable of performing tasks that typically require human intelligence, such as reasoning, decision‐making, or language understanding. Within AI, ML is a subfield that uses statistical algorithms to enable systems to learn patterns from data and make predictions or decisions that generalize well to new, unseen data.^127^ Given the current state of the field, we will use the term *ML* in our remaining discussion for greater accuracy. Key ML milestones are provided in Figure 2C’s timeline for reference. Neural networks first emerged in the late 1950s, with Rosenblatt using virtualized neurons in the early 1960s to build a Perceptron, a binary image classifier machine.^92^ Despite Rosenblatt's intellectual contribution, technical challenges surrounding building more complex neural networks and developments in other CV techniques such as edge detection largely left neural networks dormant until convolutional neural networks (CNNs) emerged in the late 1980s and demonstrated strength in finding patterns within digital images despite differences in position and orientation.^93^ ^,^ ^128^ ^,^ ^129^

ML researchers recognized the need for large, well‐labeled datasets to accelerate model development, culminating in the creation of the ImageNet dataset in 2009. This resource led to an annual competition spurring rapid progress in image classification and object localization.^130^

A breakthrough occurred in 2012 when AlexNet, a deep CNN architecture, was shown to be computationally feasible to train deep neural networks on large datasets due to its use of graphics processing unit (GPU) acceleration, ReLU activations, and dropout for regularization.^131^ This achievement marked a turning point in the practical application of CNNs and set the stage for further innovations in large neural networks denoted as DL. Additional notable DL networks for CV tasks, such as generative adversarial networks (GANs), emerged in the 2010s, demonstrating models could learn representations of objects and generate images comparable to a model's input data.^93^

In the 2010s, DL demonstrated significant promise in solving many CV problems, but medical imaging application development remained difficult given data complexity and the lack of openly accessible, well‐curated data sets.^132^ Likewise, early ML efforts in medical imaging were largely focused on radiology, where initial attempts at image classification and object localization showed limited success.^133^ Trained models often struggled to generalize across different datasets, underscoring challenges such as overfitting to the complex and variable nature of medical imaging data, as well as the difficulty of assembling sufficiently large and well‐annotated datasets for DL.^134^ ^,^ ^135^ Most recently, transformer‐based architectures have dominated DL, finding broad applications in and outside CV despite being initially developed in 2017 for machine language translation.^94^ Combined with significant advances in DL computer hardware, transformers demonstrate promise in solving medical domain problems, allowing models to use “attention” to interpret more complex patterns embedded within medical images (Figure 2D). Unlike their predecessors, transformer‐based models demonstrate significant improvements in medical imaging‐related tasks, but generalizability in medical imaging remains difficult given noise and variability common in medical data, as well as pre‐analytic variables due to the heterogeneity of equipment and software used to acquire the data.^136^ ^,^ ^137^

In general, popular CV DL models are developed using images of a lower resolution than that seen in typical medical images, creating technical challenges in scaling popular approaches to medical images. Under the premise that transformer models with similar designs could solve a larger scope of problems, researchers turned their attention to developing foundation models that featured core architectures that output core data features (encoding) for downstream models that would utilize those features in different CV tasks (decoding) in the hope that using such a model for different tasks would minimize data labeling and retraining.^138^, ^139^, ^140^ To deal with larger data sources like WSIs, various approaches have emerged attempting to encode positional information with transformer model outputs for downstream decoding that integrates “patches” together to generate outputs from larger collections of inputs (Figure 2E).^141^ ^,^ ^142^ Unfortunately, such approaches still depend on computational connections between patches requiring larger hardware memory, a major hardware and computational cost determinant. Combining such approaches leads to a significant improvement in CV tasks using complex and massive resolution image data found in WSIs. Within pathology, applications for vision transformers (ViT) and their derivatives, such as distillation of knowledge with no labels (DINO) version 1 and 2, are becoming common foundation model architectures employed in medical imaging research.^95^, ^143^, ^144^, ^145^, ^146^, ^147^

Collectively, these technologies show promise for studying neurodegeneration and clinical neuropathology tasks with technical challenges surrounding integrating data from multiple WSIs generated from different staining protocols and brain anatomic regions.

### 3.6 Adoption challenges

A multitude of different technologies exist to support using digital pathology in neurodegeneration research; the choices made from these options will depend on research group resources and personnel core strengths. Currently, technical challenges exist in WSI storage, access, and transfer with notable net benefits from using laboratory and institutional onsite infrastructures. Academic institutions commonly favor developing their own infrastructure and investing in engineering and research personnel to manage and develop shared research resources.^50^ In a survey from 2019 sent to AD research centers, 50% of respondents stated they received institutional support for scanner purchasing and operations.^33^ Utilizing open‐source frameworks and tools can solve many common challenges in infrastructure development, but associated cost savings depend on hardware investment and software engineering support. Commercial products remain viable options for research groups weary about investments in hardware and personnel; cloud platforms can be expensive for the large amounts of data generated by large‐scale WSI slide scanning. For example, using Amazon standard S3 to store 100 4‐GB WSI images (400 GBs) and accessing those data once daily for model training in a year (146,000 GB data transfer) amounts to around $7309 based on an online pricing calculator (last calculated on August 27, 2025).^148^ Additionally, proprietary data formats and tools often create challenges in accessing relevant analysis input and output data, making such tools difficult to share and validate. Building AI models often requires complex and costly computer hardware and software. For example, pathology foundation models require a specialized data science‐focused accelerator hardware, such as NVIDIA's L40S (∼$10,000) or H200 (∼$30,000), making a premier 8‐GPU server cost around $260,000.^149^ Although progress in DL will likely improve accessibility, acquiring the needed infrastructure or accessing it through a cloud provider can be costly and require considerable investment from research groups.

### 3.7 Experts’ role

Insights into neurodegenerative diseases will benefit immensely having domain experts working closely with computational researchers to address challenges unique to neurodegenerative digital pathology data. Research and development in digital pathology and ML models should involve significant contributions from experts in the field of neurodegeneration, as has been done with recent advancements.^126^ ^,^ ^150^ Continuing the progress in ML will benefit from developing comprehensive and well‐curated digital pathology datasets with well‐structured metadata, rigorous metrics for evaluating ML model performance in common neuropathology use cases. Practical insights into subtle neuropathology digital pathology data properties will be useful to understand as they could shape model biases, limiting their generalizability.^151^ Moreover, expert participation could enable more sophisticated processes for creating relevant training data through interactive processes with model outputs.^152^ ^,^ ^153^

## 4 PROGRESS IN DIGITAL PATHOLOGY RESEARCH IN ADRD

### 4.1 Summary of current diagnosis

Neurodegenerative diseases manifest clinically as movement, cognitive, and/or behavioral disorders often correlated with macroscopic anatomic changes in brain radiological images.^154^ This complex collection of findings requires comprehensive neurological evaluation given that often persons who suffer from these devastating diseases manifest with mixed clinical pictures involving a complex interplay between neuropathological processes occurring over a person's lifetime.^155^ These complex overlapping pathological processes make pathologic analysis at autopsy the current gold standard for the diagnosis of neurodegenerative diseases given no single or combination of clinical and/or radiological features is specific for a neurodegenerative disease.^156^

Neurodegenerative diseases are defined pathologically by their selective vulnerability of regional or cell type involvement.^156^ Progressive neuronal dysfunction and death are associated with specific intracellular and extracellular protein aggregates within the brain^157^ related to abnormal autophagosome/lysosomal proteolysis,^158^ oxidative stress,^159^ programmed cell death,^160^ and/or cell responses interpreted as neuroinflammation.^161^ For example, AD is classified as a neurodegenerative proteinopathy characterized by aggregates of tau in the form of neurofibrillary tangles (NFTs), neuropil threads, and neuritic plaques and aggregates of Aβ in the form of amyloid plaques.^156^ ^,^ ^162^

Despite emphasizing protein aggregates in disease classification, neurodegenerative diseases also demonstrate overlap in combinations of pathologic features that may vary in their anatomic involvement. Therefore, the diagnostic procedure at autopsy requires sampling tissues from multiple anatomic brain regions and using staining techniques such as H&E as well as multiple immunohistochemistry (IHC) markers (Figure 1D). Experts can use a collection of stained specimens from the same person and apply an established staging system, such as the Braak NFT staging system,^163^ to describe sequential distribution of misfolded protein in the brain. However, some AD subsets do not necessarily fit within the Braak NFT distribution staging system.^164^ Likewise, the Unified Staging System for Lewy Body Disorders (USSLBD)^165^ and TDP‐43 proteinopathy staging systems^166^ exist as counterparts for other neurodegenerative diseases and may face challenges similar to those faced by the Braak NFT staging system with respect to generalizability. Given emerging research and the delineation of other neurodegenerative diseases, the criteria for AD diagnosis evolved to include neuritic plaque semiquantitative methods, Consortium to Establish a Registry for Alzheimer's Disease (CERAD),^167^ and topographic distribution of amyloid plaques by Thal et al.^168^ Criteria and further delineation among neurodegenerative diseases is likely to continue to evolve as developments in technology and methodologies emerge enabling a more comprehensive neurodegenerative assessment at autopsy.

### 4.2 Quantifying histopathologic disease burden

A major goal in digital neuropathology neurodegenerative disease research is to develop scalable robust reproducible approaches to quantify histopathological features in WSIs and understand how such features contribute to disease burden. Developing quantitative methodologies and techniques that combine pathologic feature identification with statistical approaches to build associations with other available data (imaging, clinical, demographics) could shape our understanding of neurodegenerative disease pathogenesis, enable therapeutic intervention evaluation, and guide future therapeutic approaches. Moreover, creating scalable approaches that can quantify a large collection of pathological features could provide powerful multidimensional data demystifying complex interactions between different neurodegenerative processes with the aim of understanding their collective impact on disease morbidity and mortality. Quantifying histopathologic features in WSIs begins with a task referred to as object detection. Object detection tasks can involve object localization with classification or segmentation, a definition of specific pixels within the image belonging to a desired object (Figure 2B).^169^ Several open‐source histological segmentation software programs and plugins exist, with some implementations designed for ease of use in popular open‐source image analysis platforms.^170^

**FIGURE 3:**
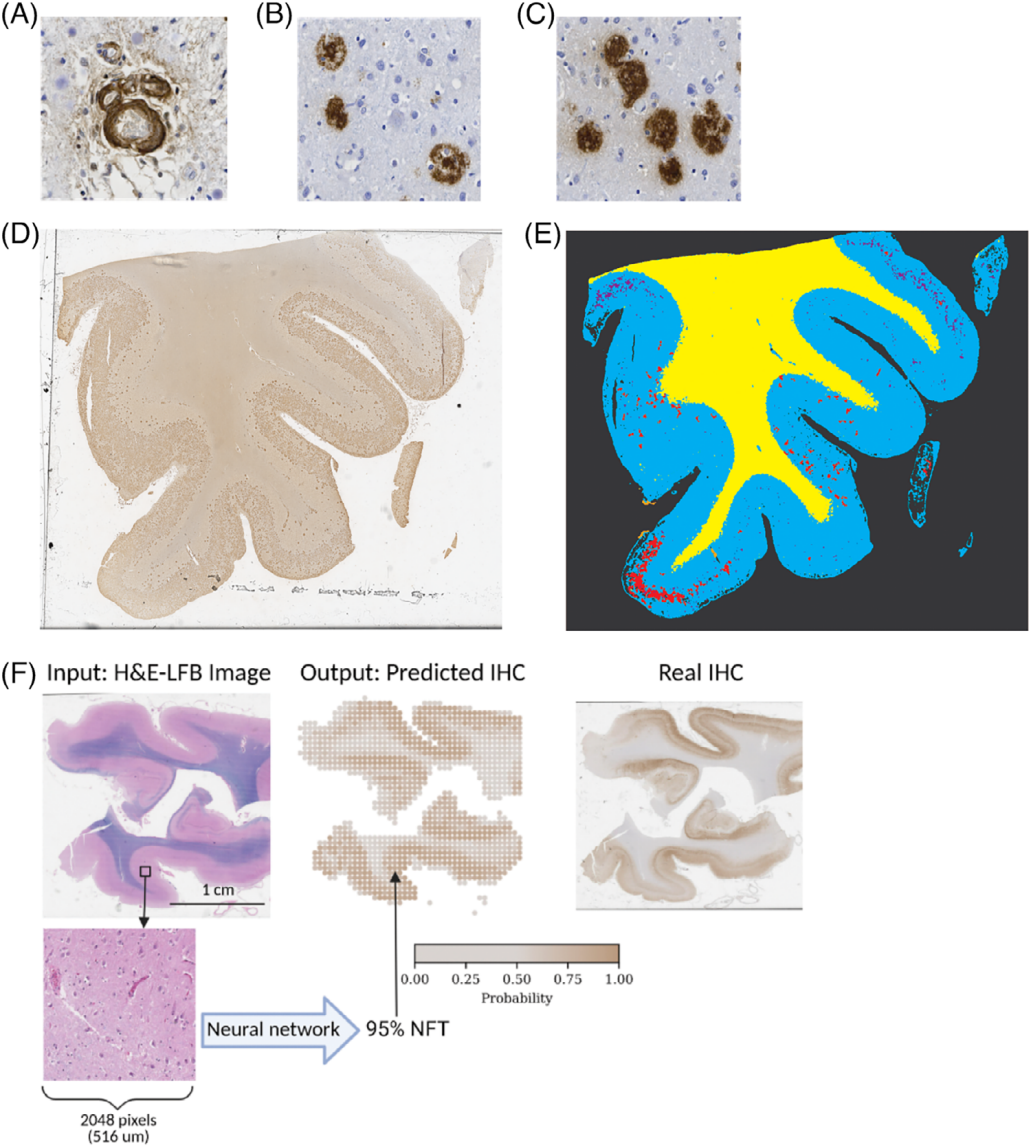
Machine learning for research in studying neurodegenerative disease using digital neuropathology. (A) Cerebral amyloid angiopathy (CAA), (B) cored plaques, and (C) diffuse plaques in a neuropathology specimen immunohistochemistry (IHC) stained for amyloid beta deposits using 4G8 antibody. (D) Visualization of whose slide image (WSI) stained using 4G8 antibody for amyloid beta deposits, from an IHC stained neuropathology specimen scanned by an Aperio AT2 at 40× magnification featured in Tang et al. 2019.^90^ (E) WSI processed using BrainSec pipeline in Lai et al. 2022 to show detailed automated annotations and masks for grey (blue) and white (yellow) matter regions with CAA (orange), cored plaques (red), and diffuse plaques (purple) that can be used to quantify pathologies and identify key relationships between pathological densities in grey versus white matter and other clinical or biological factors.^172^ (F) Example of using neural networks to re‐represent hemotoxylin and eosin (H&E)‐Luxol fast blue (LFB)‐stained neuropathology specimen predicting IHC staining showing how existing imaging data can be reinterpreted for detection‐related tasks. Image obtained from portion of figure in He et al. (2022) with reprint rights obtained using Copyright Clearance Center (Order No.: 6011480132872).^179^

Digital pathology enabled Aβ plaque quantification, in human *post mortem* samples, and showed concordant increases in histopathologic features, Braak NFT stage, and Thal amyloid phase^109^ ^,^ ^171^ providing motivation for further ML model development. Published ML models currently exist for NFT detection and segmentation,^89^, ^91^, ^172^ various plaques,^90^ gray and white matter segmentation (Figure 3E),^173^ microinfarct screening,^174^ arteriolosclerosis,^175^ and other histopathologic features, with utilizing fully convolutional networks,^172^ ^,^ ^176^ CNNs,^177^ You Only Look Once (YOLO) versions 3^88^ and 5,^89^ U‐Net,^177^ and Google AutoML models.^178^ Published models vary in their dataset sizes, which involves labeled tiles within WSI collections ranging from five to 134 files and 200 ROIs to 360,000 tiles in papers published between 2019 and 2023. Generally, more advanced models with larger, well‐designed datasets demonstrate improved performance directly on a specific task and/or better enabled downstream applications. Notably, Vega et al., Manouskova et al., and Vizcarra et al. demonstrated model compositions to enable disease classification, segmentation, and Braak NFT staging, respectively.^89^ ^,^ ^176^ ^,^ ^177^ These publications show how ML applications integrating multiple models can solve more complex problems in the neurodegeneration pathology subject domain. One must be cautious when applying published ML algorithms to their WSIs, as pre‐analytic variables (such as storage format, compression rate, and magnification) can alter results.^151^

### 4.3 Correlating histopathologic findings with preclinical disease and clinicopathologic features

Neurodegenerative diseases present with overlapping phenotypes, frequent mixed pathologies, and limited clinical and/or radiologic specificity – leading to misdiagnoses, even from expert diagnosticians.^180^, ^181^, ^182^ These challenges persist despite advances in neuroimaging and expansion of cerebrospinal fluid (CSF) and blood‐based biomarkers, which are helpful tools but still incapable of consistently diagnosing mixed or atypical dementias.^183^ ^,^ ^184^ Additionally, these newer imaging and biomarker assays are also not universally available to all patient populations. Therefore, definitive diagnosis of specific lesions still requires *post mortem* brain autopsy assessments, limiting our ability to resolve clinicopathologic uncertainty during life.

Many neurodegenerative disease WSIs are generated from human *post mortem* brain autopsy samples from brain biorepositories or other research studies. These brain banks collect and store biospecimens donated from research participants who underwent longitudinal clinical evaluations, brain imaging studies, and CSF/blood‐based biomarker assays performed for many years prior to death.^185^ ^,^ ^186^ This provides a rich multimodal data archive linked to histopathologic data. Taken together, this creates a natural substrate for ML models that can create maps between clinical data, imaging data, and biomarker data to tissue‐level pathology, with the goal of identifying and delineating specific lesion patterns during life to improve diagnosis and clinical decision‐making.

McKenzie et al. used WSIs from formalin‐fixed paraffin‐embedded frontal cortex and hippocampus with clustering constrained attention MIL (CLAM) with ResNet‐50 to extract tissue features followed by HistomicsTK deriving a LFB‐based intensity score that predicted mild cognitive impairment and correlated with age at time of death, Braak NFT stage, and vascular pathology burden.^187^ Marx et al. similarly combined ResNet‐50 with CLAM in a multiple instance learning (MIL) framework (HistoAge) to estimate brain age from *post mortem* H&E‐LFB‐stained brain autopsy slides.^188^ Together, these studies demonstrate how model compositions leveraging weak supervision can produce quantitative slide‐level metrics that align with complex clinicopathologic endpoints.

Building on these results, models integrating clinical trajectories, neuropsychological test scores, neuroimaging findings, and fluid biomarker measurements could potentially generate individual‐level probabilistic estimates of underlying histopathologic signatures (including mixed brain pathologies) – providing an enhanced foundation for precision medicine approaches. Models have the potential to effectively triage patients for confirmatory testing or clinical trials, track treatment response, and quantify uncertainty to determine when human adjudication is required. In the future, foundation‐model approaches may improve generalizability across institutions, provided they are prospectively validated and calibrated to mitigate pre‐analytic and cohort specific differences. We view these models as augmenting – not replacing – neuropathologic gold standards, while enabling earlier and more accurate decision‐making during life.

### 4.4 Extending neurodegenerative disease phenotyping

The anatomic location of neurodegenerative disease pathological features shape clinical presentation and disease characteristics. Unfortunately, the burden of a comprehensive analysis that identifies all neurodegenerative histopathologic features, their microanatomic locations, and their location relative to other histopathologic features precludes potential neuropathologic analysis insights. ML techniques display potential to dramatically increase the amount information obtainable from WSIs, enabling neurodegenerative researchers to extend disease criteria and severity scaling tools, an approach that could empower precision medicine approaches for neurodegenerative disease spectra. The first challenge is to train models to recognize differences and similarities between histopathologic features and demonstrate those findings correlate with dysfunction and disability. Tang et al. demonstrated a segmentation model could distinguish between image tiles with cored plaques (Figure 3B), diffuse plaques (Figure 3C), and CAA (Figure 3A) in Aβ IHC‐stained images;^90^ this highlights segmentation models can capture information about distinct morphological features, with different anatomic distributions, interpretable features, and CERAD‐like manual scoring correlates with subsequent studies relating these data to clinical, genetic, and pathologic findings. Likewise simpler techniques exploring morphological differences in stained samples can also yield insights. Kim et al. performed analysis using specific regions of interest (ROIs) and image thresholding to select for glial cytoplasmic inclusions (GCIs) in α‐synuclein‐stained IHC samples and subsequently utilized K‐means clustering with ImageJ size, density, and quantity measurements to discover variability between GCIs in the putamen and cerebellar white matter.^189^ Additionally, neurodegenerative pathology researchers can potentially leverage radiologic models to uncover heterogeneity in neuropathological data. Mastenbroek et al. utilized the ordinal Subtype and Stage Inference (SuStain) model with semi‐quantitative data generated from Lewy‐type alpha‐synuclein (LTS) density characterizing disease severity in 10 brain regions to identify three spatiotemporal Lewy body pathology trajectories and subsequently demonstrate correlations with age at death and USSLB staging.^190^ Advancements in ML‐assisted analysis tools will provide neurodegeneration researchers with powerful research tools to more effectively utilize WSI research data.

### 4.5 Novel detection methods

ML approaches demonstrate the potential to identify complex patterns within data that would otherwise be difficult for humans. ML models can also transform input data into useful representations that allow humans, or other computers, to interpret the data in novel ways. He et al. 2022 utilized H&E‐LFB, a stain commonly used to highlight white matter in the brain, with corresponding IHC images staining for Aβ and phosphorylated tau to predict IHC staining from H&E‐LFB images (Figure 3F). Interestingly, their approach demonstrates an impressive IHC representation achieving areas under the curve of 0.92, 0.90, and 0.92 for random H&E‐LFB image patches featuring NFTs, neuritic plaques, and Aβ plaques, respectively.^179^ Such research suggests the potential for utilizing WSIs generated from specific staining protocols to predict different stains, with neuropathologic significance potentially extending the usefulness of existing images from different brain regions or comparing brain WSIs generated by autopsies that did not formally undergo neurodegenerative diagnosis processes. DL model techniques applied to WSIs could create additional methodologies for detecting relevant neurodegeneration histopathologic features, further extending WSI usefulness to answer neurodegeneration research questions.

### 4.6 Accelerating dataset development

ML model development relies heavily on the characteristics of input training data, but input data in many medical imaging domains for tasks such as object localization and segmentation can differ based on the individual annotating and labeling the data. Wong et al. utilized Aβ‐stained IHC WSIs from three different institutions and labeled by five experts for cored plaques, diffuse plaques, and CAA to explore how differences in labeled data influence trained CNN model performance. They demonstrated training with their combined dataset enhanced model performance over models trained from individual experts.^191^ Their findings demonstrated how DL models can integrate knowledge from different experts, but developing such datasets requires significant person‐hours from experts conducting labeling and ML researchers who refine datasets and model training. Experts need scalable and practical tools that enable rich dataset generation to overcome common challenges in developing ML models for studying neurodegeneration. ML research methods less dependent on dataset labeling and refinement are commonly referred to as semi‐supervised or unsupervised methods.^192^, ^193^, ^194^, ^195^ Lai et al. developed a semi‐supervised active learning framework that can iteratively select annotation regions within WSIs to efficiently generate a labeled dataset that improved white and gray matter segmentation.^196^ Developing similar approaches for generating useful neuropathological datasets will further accelerate ML model development potentially creating well‐designed datasets for neurodegeneration investigations.

### 4.7 Exploring immune and inflammation contributions to neurodegeneration

Neuronal injury causes inflammatory processes mediated by resident and infiltrating immune cells. Central nervous system features of specialized resident immune cells, such as glial cells and astrocytes, play important roles in development and homeostasis. Likewise, ADRD demonstrate immune‐associated genetic risk factors influence soluble factors and cell surface receptors that shape neuroinflammation.^197^ Because known immunological and inflammatory processes occur in neurodegeneration, Murray et al. examined microglial activation in *post mortem* brain samples acquired from HIV‐positive individuals by utilizing QuPath for CV‐based analysis of IHC microglial activation markers. They found differences in microglial activation for patients with undetectable and detectable HIV disease as well as correlated microglial activation with cognitive testing.^198^ Munoz‐Castro et al. conducted a similar analysis on microglia and astrocytes using activation marker cyclic multiplex fluorescent IHC coupled with ML‐based methods (spectral clustering, gradient boosting machine, and CNN) to quantitatively characterize AD brains and reveal distinct microglial and astrocyte phenotypes. Consequently, their distinct microglial and astrocyte phenotypes identified a novel intermediate phenotype distinguished between normal aging and AD.^199^ Bathe et al. analyzed IHC‐stained human *post mortem* brain samples with QuPath to determine area percentages for pathological and microglia staining as well as quantify IHC‐positive cells to compare AD, Lewy body disease (LBD), and AD/LBD mixed pathologies within hippocampus, frontal cortex, occipital cortex, and substantia nigra samples. Their quantitative analysis allowed for the examination of microglial activation and phenotype, motivating additional analyses with RNA quantification and WSI deep spatial profiling to extend their immunological findings. They determined extracellular protein pathologies correlated significantly with microglial activation and that intracellular pathologies caused more subtle and global immune responses.^200^ Collectively, progress in studying immune and inflammatory contributions to neurodegeneration highlight digital pathology's potential in identifying immunological contributions to neurodegenerative disease pathogenesis, possibly revealing targets for therapeutic development. Likely, additional research exploring immunological processes will show important distinctions between disease processes and support efforts in developing preclinical indicators and extending disease phenotypes.

## 5 FUTURE OF DIGITAL NEUROPATHOLOGY

### 5.1 State‐of‐the‐art neurodegeneration digital pathology

Digital neuropathology integration into ADRD workflows will likely vary among institutions based on their needs and available resources. A 2019 survey sent to ADRC digital pathology infrastructure indicated Aperio and Phillips instruments were the most commonly deployed scanners, with varying institutional practices for sample collection, staining, and labeling.^33^ As ADRCs increasingly leverage ML in digital pathology research, they will benefit from enhancing harmonized processes for collecting and storing brain bank research emphasizing practical aspects surrounding sample acquisition, staining, and imaging, as well as linking associated clinical data such as neuroradiology, cognitive testing, and genetic data. Neurodegeneration sample preparation and imaging can follow guidelines such as those offered by the College of American Pathologists (CAP).^201^ Once high‐quality WSI data are uploaded to an image management platform, an expert can remotely access these data to complete a workflow like a standard microscope system. An image management platform with integrated ML/AI tools can augment expert capabilities by identifying areas with notable histopathologic features, simplifying annotation, streamlining data entry, and assisting report generation. Neurodegeneration researchers can further customize their tools for improved workflow efficiency and specific research questions.

### 5.2 Infrastructure modernization

Emerging CV techniques combined with ML processes demonstrate significant potential to improve neurodegenerative disease diagnostic workflows, but neurodegeneration research will also greatly benefit from increasing available WSI data and analysis tool accessibility. Likewise, institutions must make decisions regarding WSI infrastructure investments. Hospital and research digital pathology transitions will require substantial investments in slide scanners, data storage solutions, and platforms for accessing and analyzing WSI data. Neurodegenerative disease researchers working at institutions transitioning to digital pathology should be actively involved in purchasing discussions, as this can leverage and enhance research resources. Publications documenting investments in infrastructure suggest neurodegeneration researchers will benefit from onsite infrastructure for data storage and computation given the difficulties associated with transferring and storing WSIs using cloud service providers will likely be costly and impractical for large‐scale use.^50^ Moreover, it remains essential researchers retain user‐friendly access to data created during analysis in useable formats for consumption by commonly used visualization and analysis platforms. Some have published benchmarks in select fields characterizing equipment, personnel, and software needed to transition diagnosis workflows and research into modern digital pathology infrastructure.^202^, ^203^, ^204^, ^205^ Digital pathology in‐house ML research infrastructure will likely benefit from recent research‐focused hardware releases such as the Nvidia RTX Pro 6000^206^ and Nvidia DGX,^207^ but hardware with the highest memory capacity necessary for building state‐of‐the‐art complex models will continue to come at a premium.

### 5.3 Cross‐institutional research collaborations

High‐quality data repositories enhance research productivity and quality, but neurodegeneration researchers often lack resources, including expertise needed for creation, validation, and testing to overcome common medical imaging challenges. Cross‐institutional research collaborations would benefit neurodegeneration research by increasing data availability, improving the generalizability of findings/methods, increasing available datasets, and enabling more cooperation on difficult neurodegeneration research questions. In addition to creating larger datasets, cross‐institutional datasets will likely feature WSIs from different slide scanners, staining protocols, and cohorts having different demographics and recruitment criteria. This was done previously in AD neuropathology research.^89^ ^,^ ^191^ ^,^ ^208^ In ML research, heterogeneous datasets play an important role in reducing problematic biases that hinder model performance and generalizability.^209^ ^,^ ^210^ Likewise, cooperative research will create opportunities to utilize outside expertise to create productive workflows that enhance quantitative capabilities and utilize ML models in novel compositions to address complex research questions. The BDSA^86^ seeks to create a large brain WSI repository for neurodegeneration research containing WSIs from multiple institutions. The BDSA initiative will address challenges associated with sharing large WSI datasets among institutions and standardizing metadata useful in neurodegeneration research. The BDSA will accelerate modern ML neurodegeneration research applications and create unique opportunities characterizing histopathological features and correlating such features with data collected during life.

### 5.4 Hospital/hospital system middleware

Ideally, digital pathology institutions will utilize or develop software infrastructure that will work with validated commercial platforms to streamline WSI‐based research and development, as was done in radiology. Despite available commercial slide scanning solutions validated for primary diagnosis in surgical pathology, generating high‐quality WSIs requires processes that identify problematic artifacts, sample illumination, or abnormal optics that complicate their use for diagnosis and research.^32^ Although commercial platforms may emerge that aim to tackle WSI quality issues, heterogeneity in staining techniques, and sample characteristics, there may be additional challenges to adopting such technologies for neurodegeneration research, as prior sample preparation and collection may make adopting harmonized protocols infeasible since they would negate previous collections. Neurodegenerative research groups can invest in middleware to build systems that ensure WSI image quality, minimize storage needs, and maximize their utility. Specifically, middleware can solve interfacial problems between key digital pathology technologies and applications.

### 5.5 The future of ML for neurodegenerative disease in neuropathology

Previously discussed applications highlight the utility of ML in addressing important neurodegeneration research questions, but they remain underutilized in neurodegeneration. A significant reason for WSI underutilization stems from a lack of large well‐annotated and easily available curated datasets. Emerging techniques offer designs that can learn useful WSI features from more limited training datasets or potentially without well‐annotated data provided researchers adopt careful strategies for picking useful training data. Transformers^94^ will become increasingly common in digital pathology research and in histopathological feature detection within neurodegeneration helping researchers overcome dataset limitations. Additionally, neurodegeneration ML research will also likely focus on clever methodologies for developing training data. Outside of neurodegeneration, digital pathology ML advancements highlight novel model combinations featuring YOLO for tumor microenvironment feature extraction,^211^ digital pathology‐specific large language and large multimodal models, as well as foundation models.^95^ ^,^ ^143^ ^,^ ^212^ ^,^ ^213^ More recent developments in digital pathology combining modern ML methodologies reflect the tremendous progress that has been made in medical imaging applications. Moreover, the latest advancements demonstrate interesting strategies for dataset development and integrating features across WSIs. For example, Xu et al. Prov‐Gigapath demonstrate state‐of‐the‐art performance in multiple cancer‐related digital pathology tasks by generating a novel vision transformer, GigaPath, for pretraining, and Prov‐GigaPath, a combination of their vision transformer with LongNET, another recently developed transformer model to integrate slide‐level features together.^95^ We expect neurodegeneration researchers will also create similar models adapted for neuropathology use cases and hopefully expand information integration across multiple WSIs for neurodegeneration research‐related tasks.

## CONFLICT OF INTEREST STATEMENT

The authors have no conflicts to interest to disclose.

## Supporting information




Supporting Information
